# Foraging Activity of Honey Bees (*Apis mellifera* L., 1758) and Exposure to Cadmium: a Review

**DOI:** 10.1007/s12011-024-04118-3

**Published:** 2024-03-05

**Authors:** Stephane Knoll, Maria Grazia Cappai

**Affiliations:** Institute of Animal Productions of the Department of Veterinary Medicine, University of Sassari, Via Vienna 2, 07100 Sassari, Italy

**Keywords:** Cadmium, Honey bee, Contamination, Pollution, Toxicity

## Abstract

Honey bees are commonly exposed to a broad spectrum of xenobiotics, including heavy metals. Heavy metal toxicity is of concern in the context of global pollinator declines, especially since honey bees seem to be particularly susceptible to xenobiotics in general. Here we summarize current knowledge on the interplay between cadmium, one of the most toxic and mobile elements in the environment, and honey bees, the primary managed pollinator species worldwide. Overall, cadmium pollution has been shown to be ubiquitous, affecting industrial, urban and rural areas alike. Uptake of this heavy metal by plants serves as the primary route of exposure for bees (through pollen and nectar). Reported cadmium toxicity consists of lethal and sublethal effects (reduced development and growth) in both adult and larval stages, as well as various molecular responses related to detoxification and cellular antioxidant defence systems. Other effects of cadmium in honey bees include the disruption of synaptic signalling, calcium metabolism and muscle function.

## Introduction

The honey bee (*Apis mellifera* L*.,* 1758) is an economically significant domestic insect valued for numerous products including honey, beeswax, pollen, propolis and royal jelly [1]. Additionally, the ecosystem services provided by these animals in terms of pollination of wild plants and agricultural crops are arguably even more important [2, 3]. Bees contribute through these services to the preservation of plant biodiversity and consequently all associated organisms higher up in the food chain. Likewise, pollinators play a key role in agricultural sustainability and food security in a modern climate [4–6]. However, a decrease in economic gain, stability of pollination and bee populations has been described across the Western world [4, 7]. In the last decades, wild and domestic pollinators have been experiencing severe declines, raising considerable concern within the scientific community, as well as in the rural sector (agriculture and beekeeping) [6, 8–15]. Although the multitude of interacting factors behind these losses are far from being fully understood, bee declines have been coupled with the increasing effects of pests and diseases (e.g. the ectoparasitic mite *Varroa destructor*), pesticide use, climate change, feed shortage (melliferous plants),and the intensification of agricultural practices causing habitat and forage biodiversity loss [9, 11, 16, 17]. In this complex background, environmental contamination, including heavy metal pollution, is believed to have significant consequences for bee health, contributing to these declines [3, 15, 18, 19].

Since the late nineteenth century, increasing anthropological activities related to industrial, agricultural and urban outputs have caused a steep increase in environmental heavy metal burdens around the globe. This, in combination with the fact that heavy metals do not decompose, has led these contaminants to commonly be found in the atmosphere, soil and water, as well as in numerous organisms after entering biological cycles [18, 20–22]. In general, honey bees are exposed to mineral elements through food and water, and this is no different for heavy metals [15]. Accumulation of heavy metals has previously been shown in plants, including in nectar and pollen, the main feed recourses gathered by honey bees [22–28].Other routes of contamination for bees include the inhalation of contaminated airborne particles and adhesion to their hairy exterior from soil, plants and atmospheric deposition [29–31].

Though metals like copper (Cu), zinc (Zn) and selenium (Se) are crucial trace elements for insect metabolism, other elements like cadmium (Cd), lead (Pb), mercury (Hg) and arsenic (As) have no known physiological function within the insect body [15, 31]. The latter are believed to be toxic even at low concentrations. Overall, toxic heavy metals interfere with biological processes through interaction with macromolecules and/or replacing/affecting the function of essential elements in other ways [15, 18, 32, 33]. In insects, heavy metals have been reported to cause cellular structural and genetic damage among others, potentially disrupting cell functionality and causing apoptosis and mutations. Furthermore, negative effects on insect survival, development, growth and reproduction have been pointed out [22].

While the specific adverse effects of various heavy metals on pollinators are still largely unknown [19], recent efforts have been undertaken to define the effects of toxic metals on honey bee development and survival, including from a physiological and biochemical point of view [18, 23, 34–41]. Furthermore, heavy metals in bees and their products, as well as the interplay with environmental contamination, have received considerable attention [5, 42–54]. Lastly, the potential role of honey bees and their products as bioindicators for toxic metal pollution has been highlighted [14, 15, 29, 30, 39, 45, 55–66].

**Fig. 1:**
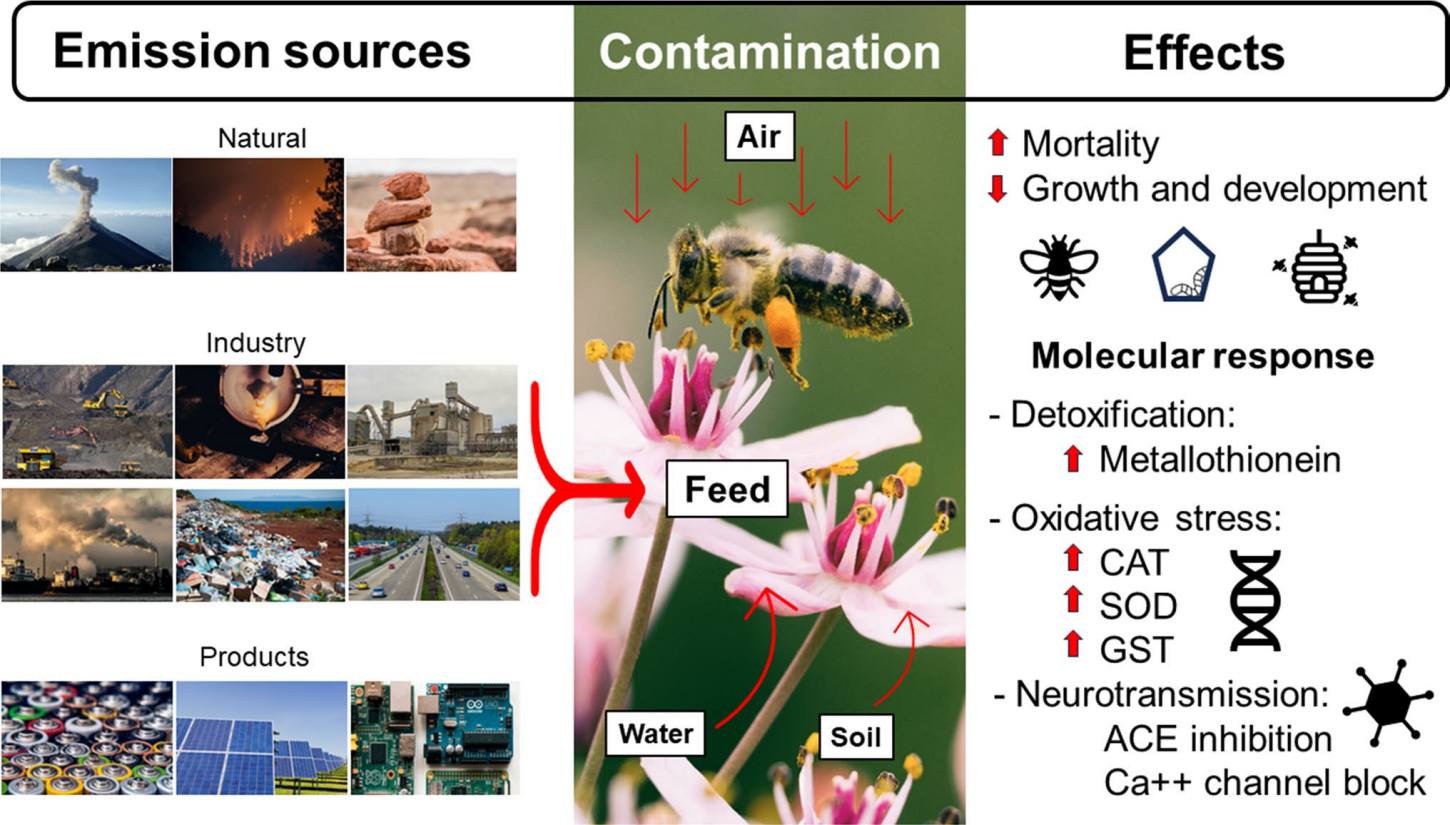
The main Cd emission sources, contamination routes for bees and the effects of cadmium on honey bees

In light of this, this review outlines the interrelation between honey bees and Cd, one of the most toxic and mobile elements in the environment [35, 67], based on the most relevant scientific literature. This review means to centralize the current knowledge in a comprehensive manner in the hope of instigating further research and management efforts in mitigating heavy-metal-related pollinator declines. A summarizing figure of the main Cd emission sources, contamination routes for bees and the effects of this heavy metal on honey bees is shown in Fig. 1.

## Cadmium (Cd)

Cadmium is a highly toxic metal harmful to humans’, animals’ and plants’ health [68, 69]. Cadmium is naturally present in soil, the lithosphere and sedimentary rock [70] and is often found together with zinc, lead and copper ores [68]. Natural dispersion of Cd results from airborne soil particles, forest fires, volcanic activity, soil erosion and the abrasion of rocks [67, 69, 70]. Annual natural emission is reported at 1300–41,000 tons [67] and the global soil concentration of this heavy metal is estimated at 0.07–1.1 mg kg^−1^ in natural ecosystems [71]. The concentration of this toxicant in different matrices, as well as based on geographical location and/or soil type, can be found in the current scientific literature [22, 67, 68, 71].

Elevated presence of Cd in the environment is mainly accredited to anthropogenic sources linked to a wide range of human activities [69, 72]. Specifically, mining, smelting and refining of various metals, fossil fuel combustion, cement production, the use of phosphate fertilizers and municipal and sewage sludge incineration are common sources of Cd pollution. Additionally, the use of Cd in electronic devices, batteries and solar panels has led to the leaching of this toxicant from landfill sites into the environment. Lastly, industrial use of Cd as a corrosive reagent, stabilizer in PVC products and in colour pigments generates a continued source of Cd contamination [35, 67, 69–73].

Due to its specific chemical characteristics, Cd and its compounds are easily dispersed through water, polluting rivers and natural bodies of water located in the vicinity of emission sources [69, 70]. Airborne Cd can be dispersed over short and long distances and is deposited ubiquitously, including with rainwater [67, 69, 70]. Soil contamination results from the deposition of airborne particles, dispersion through water and the large variety of natural and anthropogenic Cd sources discussed above [69, 72]. As a result, Cd pollution is widespread around mining, urban, agricultural and industrial areas [69, 74] and can even affect seemingly “unpolluted” environments [15, 66]. Finally, given the bio-accumulative properties of Cd in both plants and animals (with an approximate half-life of 25–30 years), this pollutant is commonly found in a wide range of organisms including mushrooms, rice, wheat, vegetables, crustaceans, molluscs and honey bees [15, 68–70, 75–77].

## Cd Exposure of Bees

Bees are exposed to Cd mostly through contaminated plants [15, 22]. Pollen and nectar, the main feed resources collected by honey bees, have been shown to be contaminated with Cd [39, 42, 61, 78–80] and bees not to avoid contaminated feed recourses as they most likely cannot discriminate between metal-contaminated and non-contaminated plants [19, 74]. Furthermore, a dose-dependent relationship between the Cd content of bees and the concentration of this contaminant in their feed has been found [35]. Besides this, bees are exposed to Cd through inhalation and deposition of airborne particles [22, 35, 40, 45, 46, 50, 60]. Recent research comparing bee whole-body Cd concentrations with that in their haemolymph revealed significant differences (lower haemolymph concentrations) which could be accredited to the accumulation of Cd-contaminated dust particles on the bee’s hairy exterior surface [15]. External Cd in turn could be ingested by bees during grooming [22]. Alternatively, Sadowska et al. [49] report the contribution of externally adhered Cd to the whole-body content of this metal in bees to be negligible.

As Cd pollution pressure differs according to spatial and temporal variations, honey bee exposure trends are complex and highly dependent on their immediate environment [15, 22, 42, 43, 45, 46, 49, 50, 60, 61]. First of all, variations in Cd pollution pressure for bees might result from the availability of different plant resources for foraging [20–22, 78]. Indeed, Cd contents will vary substantially between plant species as well as between cultivars and genotypes of the same species [68]. This being said, limited information is currently available on the metal concentrations in the pollen and especially nectar of different plants [22].

Next, Cd contamination will vary based on the general location of apiaries, likely related to the influence of both natural and anthropogenic emission sources. In this regard, significant Cd burdens have been found in honey bees in industrial, urban, agricultural and rural areas around the globe [15, 39, 42, 43, 45, 46, 50, 53, 60, 62, 64, 68]. A recent survey analysing the Cd content of bees from various areas in Serbia revealed bees residing in the vicinity of industrial zones to be exposed to significantly higher Cd pollution as compared to those from urban or rural areas [15], as previously reported [20, 22, 48, 50]. In Sardinia, bees located near a mining area were more affected by this contaminant [44]. In Italy, Poland and Turkey, Cd contamination was greater in urban than in rural areas [29, 49, 53, 64, 66]. Substantial Cd contamination in bees from agricultural zones [15, 22, 42, 43, 45, 60, 62] and seemingly unpolluted rural areas [15, 62, 64, 66] has been reported as well which, in certain cases, was even higher as compared to industrial zones [15, 20, 60, 64]*.*

Significant temporal variations in Cd contamination of bees have been highlighted within the same study area [15, 42–44, 60]. Such variations are hypothesised to be the result of seasonal changes in dominant Cd dispersion pathways. For example, airborne Cd might represent a major contamination route during winter as climatic conditions promote atmospheric particle deposition [15]. Additionally, increased airborne Cd could be related to the elevated combustion of fossil fuels at those times [15]. Supporting this hypothesis, toxicant monitoring of deposited airborne particles by [15] revealed substantially higher Cd contents in winter, especially near industrial areas. Alternatively, seasonal variations in Cd exposure might be correlated to the state of activity of honey bees in general [15]. Overall, non-essential element concentrations in bees have been shown to be higher during spring and summer [15, 20, 44, 60] when bees are most active and thus have maximal contact with toxicants.

Lastly, significant differences in Cd pressure on a smaller time scale have been pointed out as well [45, 64]. van der Steen et al. [45] found significant variations in the Cd contents of bees from the same location over a 3-month period and argued that in highly populated and developed countries, fluctuations in anthropogenic Cd emission serve as the main factor influencing Cd pollution pressure for honey bees.

## Effects of Cd on Honey Bees

### Cadmium-Related Mortality and Altered Development

Current knowledge on the impacts of Cd on honey bee survival is still limited [23, 35, 81]. Nevertheless, lethal and sublethal effects of Cd exposure on larval and adult honey bees have been shown, even at ecologically relevant concentrations [35]. Specifically, reduced development and growth and increased mortality have been pointed out [23, 35, 81].

Cronn [81] first studied the lethal effects of Cd on adult bees and determined this toxicant to be moderate to highly toxic for honey bees. In a series of experimental trials, dietary supplementation of Cd to young nurse bees revealed a significant increase in mortality occurring as early as 24 h after initiation of the experiment. Median lethal doses (LD_50_) for oral intake of two Cd salts over various lengths of exposure (for CdCl_2_, 3.51 µg Cd/bee for 48 h, 2.80 µg Cd/bee for 96 h; for CdSO_4_, 2.34 µg Cd/bee for 48 h, 1.44 µg Cd/bee for 96 h) were reported [81]. Analogous results were published by Di et al. [35] where Cd consumption by honey bee foragers (dissolved in 50% sucrose solution) led to increased mortality both over time and with increasing dose. Furthermore, mortality was shown to increase more rapidly with increasing Cd treatment and a lethal concentration (LC_50_) of Cd of 78 mg L^−1^ was reported [35]. Other research efforts focussing on the physiological responses of honey bees to Cd revealed no lethal effects on adult individuals for supplementation ranging between 0.001 and 0.1 mg L^−1^ over a period of 2–10 days [18, 28, 31, 36].

Di et al. [35] reported the results of acute and chronic Cd toxicity tests on honey bee larvae through dosing of an artificial diet (53% W/W commercial freshly frozen royal jelly, 6% glucose, 6% fructose, 1% yeast extract, and 34% ultrapure water), showing substantial variation as compared to foragers. In this regard, authors revealed sublethal effects on larval development in addition to a dose-dependent increase in mortality [35]. Negative effects on larval growth and development were characterised by significantly lower pupal weight for animals treated with 3.16 mg L^−1^ of Cd and reduced growth rates starting from 1.05 mg L^−1^ onwards. A significant difference in mortality was seen starting from day 4 after initiation of the experiment. For the highest Cd-doses (9.47–28.41 mg L^−1^), mortality of 100% was shown after less than 1 week and some treatment groups never even reached the pupal stage. These results highlight the particularly high toxicity of Cd for honey bee larvae with a LC_50_ of 0.275 mg L^−1^ [35]. These values correspond to realistic Cd burdens for honey bees, suggesting larval survival to potentially be affected by Cd pollution under field conditions [35].

Besides these effects at the individual level, it should be considered that increased adult and brood mortality as well as altered development will likely have negative impacts on whole colony health [25, 35, 37]. However, colony-level impact studies of heavy metals, including Cd, are lacking. To the best of our knowledge, only one research investigated the effects of Cd on whole bee hives and revealed oral supplementation of environmentally realistic concentrations (through sugar syrup and pollen patties) for 60 days to have an effect on larval honey bee stages in particular [28]. Specifically, Cd treatment reduced pupal survival significantly, indicating high brood mortality to be a real threat to colonies commonly exposed to Cd. Although no difference in total worker weight was recorded, authors argue prolonged Cd exposure (exceeding 60 days) could reduce overall worker populations over time [28]. Earlier research reporting fewer adult bees and reduced productivity in hives located near heavy metal–contaminated industrial areas supports this hypothesis [82].

### Molecular Response to Cd Toxicity

Studies investigating the molecular responses of honey bees to Cd are relatively more copious. Overall, efforts have been made to identify the effects of this pollutant on detoxification and cellular antioxidant defence systems in particular [18, 28, 34, 36–39, 82, 83]. These effects are discussed together in the next paragraphs as both systems share various key aspects. Other molecular effects of Cd in honey bees include the disruption of synaptic signalling, calcium metabolism and muscle function.

#### Metallothionein

Metallothioneins (MTs) are a superfamily of metal-binding proteins present in all eukaryotes which play crucial roles in metal homeostasis and detoxification. These molecules are central for increasing metal tolerance, reducing toxic metal burdens, and are known to protect organisms against the toxic effects of metals, especially Cd [84, 85]. In fact, the initial description of MTs was as Cd‐binding proteins in the kidneys of horses [86].

Experimental research [81] first uncovered Cd-binding proteins identified as MTs in honey bees. This author reported a close to linear rise in MTs with increasing Cd supplementation and time, and honey bee mortality seemed to increase slower as compared to MT accumulation, indicating a protective function [81]. Production of such proteins was also reported in bees from Cd-contaminated areas, suggesting environmental Cd exposure to induce MT [34, 81].

The *Apis mellifera* metallothionein gene(*AmMT*) was only recently identified, characterised and sequenced and was found to code for a single protein [38]. Purać et al. [38] went on to test the Cd detoxification capacity of this protein through overexpression of recombinant *AmMT* in *Escherichia coli* revealing this to cause increased metal tolerance. Further laboratory and field experiments with honey bee workers showed a dose‐dependent relationship between Cd contamination and *AmMT* expression [38]. This was expected as Purać et al. [38] identified a metal-induced transcription factor promotion region flanking the honey bee MT gene, as previously found in other animals, including insects [87]. An increase in MT expression resulting from Cd exposure has also been reported in *Musca domestica, Folsomia candida* and *Orchesella cincta* [88–90]. Research efforts [38, 81] have provided the cornerstone for our understanding of metal homeostasis and regulation in honey bees and the potential role of MTs in metal detoxification and tolerance of Cd.

Besides their role in metal homeostasis and detoxification, MTs play an important function in cellular antioxidant defence systems. This is achieved firstly through the binding of metals preventing the production of free radicals and secondly by the direct scavenging of reactive oxygen species [84, 91]. Moreover, molecular signalling related to oxidative stress is known to induce MT expression as well [92, 93] and has been identified in honey bees [38]. Hence, Cd-induced MT expression in honey bees could (at least partly) be due to oxidative stress response.

#### Oxidative Stress

Heavy metals and their ions are known to cause oxidative stress by generating an increase in free radicals and highly reactive oxygen species [32]. Under normal circumstances, the cellular antioxidant defence system (superoxide dismutase family; SOD, catalase; CAT, among others) neutralizes these threats, but in the presence of excess amounts of toxicants (like heavy metals), defences are depleted, leading to oxidative damage [94]. Specifically, Cd is known to impede cellular antioxidant defence systems through the inhibition, depletion and/or replacement of essential elements, indirectly leading to oxidative stress [95]. The effects of Cd exposure can consequently be quantified by measuring the levels and activity of various components of the antioxidant defence mechanism, as well as the amount of oxidative damage in cells.

The effects of environmentally realistic Cd concentrations on the cellular antioxidant defence system of honey bees were investigated [18]. Authors revealed the feeding of Cd-contaminated sucrose (0.01–0.1 mg L^−1^) to adult worker bees to cause upregulation of *Cat*, *Sod1* and *Sod2* gene expressions, genes coding for the enzymes CAT and SOD respectively [96]. A linear dose-dependent increase in *Cat* and a non-linear dose-dependent increase in *Sod* gene expression was found [18], in correspondence with previous research in other invertebrates [97–99]. Upregulation of these genes shows an increased need to eliminate oxidizing radicals and thus oral Cd treatment to cause a heightened risk of oxidative stress in honey bees [18]. Additionally, an increased expression of antioxidant enzyme genes (including *Cat* and *Sod*) was found in bees from areas contaminated with heavy metals, further suggesting this to be a protective adaptation to heavy metal–mediated oxidative stress, even though contamination by Cd specifically was not investigated in these studies [5, 37].Lastly, no concrete evidence of increased oxidative damage was found by [18] after 48 h, though authors argue prolonged exposure to Cd might generate clearer results.

Follow-up research by [37] explored the effects of oral exposure of honey bees to Cd on another group of important protective enzymes. There, authors revealed, in accordance with comparable experiments in other arthropods [100, 101], a dose-dependent increase in gene expression of three classes of glutathione S-transferase (GST: Delta;*Gstd1*, Sigma;*Gsts1*, microsomal;*Gstmic1*). These are multifunctional enzymes that play crucial functions in the management of oxidative stress, including glutathione peroxidise activity and the processing of endogenous reactive intermediates and oxidative metabolites [102, 103]. Analogous to their previous findings, a more profound effect on the gene level was observed in comparison with the enzyme activity (which was not altered for GST in response to Cd), indicating gene expression to be a more accurate reflection of the acute toxic effects of Cd [18, 37]. Again, as experimental conditions only lasted for 48 h, prolonged exposure to Cd could lead to distinct results.

Lastly, α-tocopherol and carotenoid levels (well-known dietary molecules in support of cell endogenous antioxidant defence systems) do not seem to be affected by Cd supplementation in honey bees [28, 36].

#### Genetic Background

It is worth mentioning that honey bees have fewer genes coding for enzymes of cellular detoxification and antioxidative systems as compared to other insects [104]. For example, *Apis mellifera* holds approximately one-third of the genes for GST present in some other insect species [105]. Likewise, only one MT gene was found in honey bees [38], while at least two such genes have been identified in many invertebrates, with one coding for a specific Cd-binding MT [85]. Moreover, MT gene duplication in early research with fruit flies was found to double their MT production capacity leading to a heightened tolerance to metals, including Cd [81]. Consequently, *Apis mellifera* could be especially prone to effects from intoxication by heavy metals and xenobiotics in general [34, 37, 83, 105]. Alternatively, it has been speculated that other forms of defence against toxicants might be in place in honey bee colonies where caste structure, behaviour and the dilution of toxicants play a role [23, 105].

#### Neurotransmission

Previously mentioned experimental research [83] investigated the effects of Cd on acetylcholinesterase (ACE) activity in honey bees as well and showed a reduced activity of this hydrolysing enzyme in response to 0.001 and 0.01 mg L^−1^of Cd. ACE is an enzyme responsible for the metabolization of the neurotransmitter acetylcholine (in both vertebrates and invertebrates)following its release into cholinergic-type chemical synapses, including those found in neuromuscular junctions. The breakdown of acetylcholine leads to the termination of synaptic transmission and the inactivation of ACE effectively causes disruption of neurotransmission following synaptic acetylcholine accumulation and receptor hyperstimulation [106, 107]. In fact, commonly applied pesticides (e.g. organophosphorus and carbamate pesticides) specifically target and inhibit this enzyme, causing paralysis and consequentially death in insects [106, 108]. Cadmium is believed to inhibit ACE by directly binding to the enzyme, compromising its functionality either through loss of activity or deterioration [83–109].

Even though Cd is a known inhibitor of ACE, the effects of various metals on this enzyme have been shown to be species-specific [83, 109–114]. Regarding Cd and honey bees specifically, results presented by Nikolić et al. [81] indicate a clear inhibitory function on whole-body enzyme activity. While the consequences of ACE inhibition on honey bee locomotion and mortality were not explored in the mentioned research, authors do make an interesting point suggesting the effects of toxicants with a similar mode of action could be exacerbated due to additive toxicity caused by Cd. Consequently, environmental exposure of honey bees to sublethal concentrations of pesticides with synergistic effects could prove lethal in combination with Cd contamination [81].

Effects of Cd on honey bee locomotion might result from the disturbance of alternative molecular mechanisms. For instance, Cd could affect ACE activity by interfering with calcium metabolism [113, 115, 116]. Moreover, Cd has been found to block voltage-dependent Ca channels in honey bee skeletal muscle fibres, resulting in modified action potential [117]. These transmembrane channels regulating the passage of positive calcium ions are key components of membrane depolarisation and inhibition of this mechanism can therefore result in impaired neuromuscular transmission, muscle contraction and locomotion [118, 119]. This could be especially true as calcium flow is of particular importance within the mechanisms of depolarisation and muscle contraction in insects (including honey bees) compared to sodium [117, 118]. Besides, as was discussed for ACE inhibitors, an analogous additive effect of Cd on ion channel–targeting pesticides can be hypothesised [118]. One recent finding by Li and co-workers [120] linked the exposure to Cd and the depressed olfactory ability of foragers. This effect could potentially impact the localization ability of feeding source and limit the ecological role of pollination by bees. The effect of carry-over into honey [121] plays as a gatherer of interest within the scientific community not only of the effects on bee physiology and patho-physiology, playing as environmental sentinels of pollutions, but also for the role in the potential load of toxic elements into honey, as food for human consumption.

## Conclusion

The complex nature of the interplay between honey bees and environmental Cd pollution is real. While significant efforts have been put toward defining spatial patterns of contamination, research regarding Cd contents of feed (pollen and especially nectar), being the chief mode of exposure for bees, is still lacking. Realistic Cd burdens appear to represent a serious threat to honey bees in terms of development and survival and larval stages to be especially susceptible to the toxic effects of Cd. Additionally, the genetic background pointed out in this review suggests honey bees to be particularly sensitive to xenobiotics in general, including heavy metals. However, there is a need to further evaluate the effects of Cd on both adult and larval stages under field conditions. A deepening of our understanding of metal homeostasis and the molecular responses of honey bees to Cd is needed as well. Lastly, significant synergetic adverse effects between Cd and other stressors (e.g. other heavy metals and pesticides) have been accounted. In the context of understanding global pollinator and honey bee declines, investigation into heavy metal toxicity deserves continuous attention.

## Data Availability

Not applicable.
